# Toward a Clearer Understanding of Value-Based Healthcare: A Concept Analysis

**DOI:** 10.1155/jonm/8186530

**Published:** 2025-04-19

**Authors:** Vahe Kehyayan, Yasin M. Yasin, Areej Al-Hamad

**Affiliations:** ^1^College of Business, Healthcare Management, University of Doha for Science & Technology, P.O. Box 24449, Doha, Qatar; ^2^Faculty of Nursing, University of New Brunswick, Fredericton, Canada; ^3^Daphne Cockwell School of Nursing, Toronto Metropolitan University, Toronto, Ontario, Canada

**Keywords:** concept analysis, leadership, patient-centered care, patient outcomes, quality care, value-based healthcare

## Abstract

**Background:** Value-based healthcare (VBHC) aims to improve the quality of healthcare delivery while reducing costs and also aims for outcomes that are of utmost importance from patients' perspectives. Despite a growing interest in VBHC, a significant knowledge gap persists within the existing literature in the absence of a clear conceptualization of VBHC itself.

**Aim:** The aim of the present study was to develop a comprehensive understanding of the concept of VBHC in order to arrive at a definition based on the evidence in the existing literature.

**Method:** A concept analysis approach was used to identify the concept's defining attributes, its antecedents, consequences, and its empirical referents.

**Results:** The analysis of the concept yielded three defining attributes: monetary value of health service, quality of care, and patient-centered care. The analysis also identified several crucial antecedents for transitioning traditional fee-for-service models to those focused on value; it also identified key interrelated consequences: improved patient outcomes, cost reduction, and increased patient satisfaction.

**Conclusion:** The concept analysis of VBHC provides a comprehensive framework for understanding its key components and challenges. By aligning healthcare delivery with the values and needs of patients, VBHC represents a promising avenue toward achieving high-quality, sustainable healthcare. The findings from this analysis call for a collaborative effort among healthcare leaders, researchers, and policymakers to further refine and implement VBHC models, ensuring healthcare systems are both patient-centered and cost-effective. These findings also have implications for nursing management.

## 1. Introduction

Value-based healthcare (VBHC) centers on delivering outcomes that are most meaningful while optimizing costs [1, 2]. In practice, VBHC offers a structured framework for healthcare systems seeking to enhance quality and efficiency—goals increasingly critical given rising healthcare expenditures, growing chronic disease burdens, and workforce shortages [3]. By emphasizing patient-centered metrics, VBHC also aligns with global initiatives for sustainable healthcare, such as the United Nations' sustainable development goals [4, 5]. Nurses also play a crucial role in advancing the sustainability efforts within the healthcare industry [6].

A successful shift to VBHC requires effective leadership, such as transformational leadership [7]. Leaders establish organizational priorities, allocate resources, and guide policy to ensure that value-driven practices take root [8, 9]. They also play a pivotal role in coordinating stakeholders, fostering collaboration across sectors, and sustaining momentum for system-wide change [10]. For healthcare managers, understanding VBHC principles is only the first step; applying them through informed leadership strategies is crucial for achieving transformative, patient-centered care (PCC) [7].

Although the literature frequently positions VBHC as a response to escalating healthcare costs, significant gaps remain in conceptual understanding of VBHC and its practical implications for diverse stakeholders. Much of the existing literature conceptualizes VBHC goals or the broader notion of value without providing a cohesive definition of the concept itself [11]. This fragmented understanding limits the ability of policymakers and practitioners to fully leverage VBHC's potential. Therefore, a concept analysis is necessary to bring clarity to VBHC by examining its operational and conceptual dimensions, its implications for patient outcomes, and its overall effectiveness.

This paper seeks to bridge these gaps by presenting a structured analysis of VBHC. The aim is to advance a clearer conceptual understanding of VBHC and its relevance for improving both patient outcomes and healthcare delivery. By offering a more cohesive narrative, this study intends to stimulate scholarly discourse and contribute to the development of healthcare models that prioritize value-driven, PCC.

To guide our concept analysis, we adopted the framework of Walker and Avant [12]. This model is extensively employed in research to clarify ambiguous concepts [13]. The Walker and Avant framework is a systematic approach for conducting a concept analysis, and it involves eight steps: (1) selecting a concept; (2) determining the purpose of the analysis; (3) identifying all uses of the concept; (4) determining the defining attributes; (5) constructing a model case; (6) constructing a borderline and contrary cases; (7) identifying antecedents and consequences; and (8) defining empirical referents. Our analysis of VBHC followed these steps.

The first step of a concept analysis is to clearly define and select the concept for analysis. The concept selected for this study was VBHC. The reason for choosing this concept was because of its linkage to the United Nation's Sustainable health goals (Goal 3), which is to “ensure healthy lives and promote well-being for all at all ages” [5]. Healthcare managers have a pivotal role in supporting the achievement of this goal. To accomplish this, healthcare managers need to have the essential knowledge, skills, and attitude.

The second step in this concept analysis is to determine its purpose. The primary aim of this concept analysis is to elucidate VBHC within a theoretical framework and to offer a well-defined operational definition that mirrors its theoretical underpinnings, thereby clarifying and defining the concept to facilitate the development of a conceptual model. This analysis will identify and describe the extant knowledge regarding VBHC, underscoring its pivotal role in healthcare systems.

## 2. Methods

An online search of Medline and CINAHL databases was conducted using key terms such as: value-based healthcare; patient-centered care; quality of care; and leadership. These keywords were used alone or in combination using the *Boolean operators* “AND” and “OR” [14].

The following inclusion criteria were defined to guide our search of the literature: articles that were published from 2004 when publication of VBHC was first introduced in the peer-reviewed literature [15] until the time of the writing of this paper (January-February 2025). We also expanded our search by manually searching the references of several of the retrieved articles. All publications written in the English language were included.

## 3. Results

### 3.1. Identifying All Uses of the Concept

The third step in the Walker and Avant [12] approach is to describe the various uses of the concept. The terms value-based care and value-based healthcare are used interchangeably in the literature with no differences in their attributes. Both refer to the effectiveness and efficiency of healthcare. Patients desire to have quality care and a positive experience, while healthcare providers aim to have the least cost of healthcare provided without compromising patients' expectations [1]. Both aims are integral to the core of the VBHC concept.

To identify all uses of VBHC, a systematic approach was used to search the relevant literature. A search of several data sources was conducted to retrieve relevant research studies and to assess what is known on the subject and what is still unknown [16]. Dictionaries, online databases, and the gray literature were searched systematically.

#### 3.1.1. Dictionaries

Our concept, VBHC, is a complex term and was not found as a single entry in the dictionaries we consulted: Merriam-Webster and the Oxford English Dictionary. Consequently, we decided to deconstruct the term into its component parts: value, health, and healthcare.

Value is defined by Merriam-Webster [17] as “the monetary worth of something; a fair return or equivalent in goods, services, or money for something exchanged.” Alternatively, the Oxford English Dictionary [18] defines it as: “The relative worth, usefulness, or importance of a thing or (occasionally) a person; the estimation in which a thing is held according to its real or perceived worth.”

Next, we explored the term “health.” Merriam-Webster [19] defines it as “the condition of being sound in body, mind, or spirit; the general condition of the body; a condition in which someone or something is thriving or doing well.” The Oxford English Dictionary provides a slightly different definition: “soundness of body; that condition in which its functions are duly and efficiently discharged” [20].

Lastly, we examined “healthcare.” According to Merriam-Webster [21], it is “efforts made to maintain, restore, or promote someone's physical, mental, or emotional well-being, especially when performed by trained and licensed professionals.” The Oxford English Dictionary defines it as “care for the general health of a person, community, etc., especially that provided by an organized health service; frequently attributive.”

#### 3.1.2. International Online Databases

While interest in VBHC is increasing globally, driven by the sustainable development goals [5], a consensus on its definition is lacking [22]. A scoping review by van Staalduinen et al. [23] about implementation of VBHC in hospitals concluded that “the majority of the articles (included) did not specify a conceptualization of VBHC but only conceptualized the goals of VBHC or the concept of value.” Thus, common across VBHC programs, it is understood that its aim is to improve care outcomes as valued by the patient and achieved with efficient use of resources [1]. It is a blueprint for transforming healthcare systems with the primary aim of delivering patient value, which is measured as the health outcomes achieved per unit of costs; a simpler way of saying this is outcomes that matter to patients divided by cost [1, 24]. This broad characterization aligns it with the triple aim of the Institute for Healthcare Improvement (IHI)—improving health outcomes for patients, enhancing patient experience, and reducing cost [25].

According to Catalyst [26], the aim of VBHC from the patients' perspective is better outcomes and lower cost. That is, patients spend less money to achieve better health. While from the healthcare providers' perspective, its aim is to yield higher patient satisfaction rates but better care efficiencies.

#### 3.1.3. The Gray Literature

To broaden our search, we also searched the gray literature to access any sources of information that were not published in the online search databases listed above. These included governmental websites of organizations such as the United Nations, the Commonwealth Fund, and the Centers for Medicare and Medicaid (USA), and other search engines such as Google.

According to the United Nations, VBHC is defined as an approach that prioritizes the patient at its core. It focuses on fostering local ownership and is actively experimenting with innovative solutions to enhance health and well-being while reducing expenses [5]. The Commonwealth Fund [27] approaches the definition of VBHC from a different angle. It refers to it as “value-based care (that) ties the amount health care providers earn from their services to the results they deliver for their patients, such as the quality, equity, and cost of care.” The Centers for Medicare and Medicaid Services in the United States define value-based care as “a term that Medicare, doctors, and other healthcare professionals sometimes use to describe healthcare that is designed to focus on quality of care, provider performance and the patient experience” [28]. The “value” in value-based care refers to what an individual values most. Common to all of these different definitions is quality care of value to the patient and the cost of care to both the patient and the health service provider.

### 3.2. Defining Attributes

Defining attributes are those characteristics that best define a concept [12]. Of the multiple attributes that the VBHC concept may have, we determined the following attributes most suited for the purposes of this analysis: monetary value of health service, quality of care, and patient-centered care. Together, these attributes encapsulate the essence of VBHC, aligning with its goal of balancing economic sustainability with patient-centered outcomes.

#### 3.2.1. Monetary Value of Health Service

The monetary value of healthcare services within a VBHC is centered on achieving a delicate balance between cost-efficiency and patient outcomes. The economic burden of unnecessary treatments, redundant consultations, and inefficient resource utilization significantly inflates healthcare costs, affecting both individual patients and the broader system [29, 30]. Beyond direct expenditures, indirect costs—such as prolonged recovery times becaue of suboptimal care—can have equally profound economic consequences. Faster recovery not only reduces healthcare expenses for patients but also preserves workforce productivity, mitigates income loss, and enhances overall quality of life [31, 32]. Moreover, improved patient outcomes lead to long-term cost reductions, as fewer complications and a reduced reliance on prolonged treatments alleviate financial burdens at both institutional and systemic levels [24].

In addition to direct and indirect costs, avoidable hospitalizations and high readmission rates signal financial inefficiencies in care delivery. These inefficiencies highlight the need for evidence-based staffing models that optimize nurse-patient ratios and reduce the overutilization of healthcare services [33]. By ensuring that nurse staffing levels align with patient needs, healthcare organizations can reduce preventable admissions, enhance patient safety, and improve overall care quality, thereby lowering system-wide costs [33, 34].

VBHC emphasizes the premium of healthcare—the equilibrium between care quality and cost-effectiveness. Park's Sweet Spot Theory challenges the conventional assumption that increasing nurse staffing alone results in better patient outcomes [33–35]. Instead, it posits that more is not always better; the ideal balance lies within the “central optimum nurse staffing zone” (C-ONSZ), where patient safety, workforce sufficiency, and economic efficiency intersect [33, 34]. Applying this concept, nurse staffing levels should not increase indiscriminately but should be strategically adjusted to maximize health outcomes at optimal costs. This approach reduces missed care, alleviates nurse burnout, and prevents unnecessary financial strain, ensuring that resource allocation aligns with both clinical effectiveness and economic prudence.

From an organizational perspective, VBHC seeks to enhance healthcare resource utilization while rigorously assessing the efficacy of healthcare systems [36]. By linking value to measurable patient outcomes, this framework ensures that healthcare investment is outcome-driven rather than volume-based [24]. A fundamental aspect of this transformation is the integration of value-chain management principles in workforce planning. Park's model underscores the importance of shared value creation, wherein patients, providers, and financial stakeholders collectively benefit from strategic healthcare investments. This aligns with value-driven workforce strategies, ensuring that nursing resources are deployed efficiently to enhance both immediate and long-term patient well-being.

The monetary aspect of VBHC plays a pivotal role in fostering both system efficiency and PCC. By reducing direct and indirect financial burdens, as evidenced by improved clinical outcomes and optimized resource utilization, VBHC contributes to a financially sustainable healthcare model [24, 30]. As healthcare organizations strive to maximize resource efficiency and improve system performance, VBHC bridges the gap between financial viability and PCC, ensuring that healthcare expenditures are aligned with meaningful health improvements [37].

In sum, VBHC frameworks extend beyond mere cost reduction; they emphasize value-driven investments that optimize health outcomes relative to costs. When clinical improvements justify increased spending, cost-effectiveness assessments determine whether additional investments are warranted [38]. This approach aligns with VBHC's overarching goal: to maximize health benefits per dollar spent rather than simply minimizing expenditures. Achieving this requires a strategic reallocation of healthcare resources, ensuring that nurse staffing, care quality, and financial sustainability align with patient-centered outcomes. By leveraging econometric models that prioritize optimized workforce deployment, VBHC fosters a resilient, high-quality, and economically sustainable healthcare system.

#### 3.2.2. Quality of Care

Another characteristic that emerged from the literature is quality of care. Patients' perspectives on the quality of care involve a range of factors, such as the ease of access, efficacy, and interpersonal aspects of healthcare delivery. According to the Institute of Medicine (IOM) [39], it concerns with how healthcare services raise the possibility of desired health outcomes. Crucial markers of this quality are patient experience and satisfaction, which show if the treatment received meets or surpasses expectations. This attribute underscores the connection between patient experience, satisfaction, and adherence to treatment plans, all of which contribute to better health outcomes [40]. Patient experience and patient satisfaction are essential indicators of the quality of care received by patients. These measurements shed light on how well healthcare services are meeting patients' needs and expectations.

Patients' experience has been associated with their perception of care [41]. It has also been associated with patient satisfaction and PCC [42]. Several definitions of patient experience have been offered. The agency for healthcare research and quality (AHRQ) described it as patients' entire journey in healthcare [43]. Several aspects of patients' interactions with healthcare systems have been associated with positive patient experience, including access to care, communication with clinicians, getting information, shared decision-making, self management support, courtesy and respect, care coordination, and culturally appropriate care [43]. The Beryl Institute offered a definition of patient experience as “the sum of all interactions, shaped by an organization's culture, that influence patient perceptions, across the continuum of care” [44].

Patient experience is intricately linked to the quality of care, serving as both a reflection and a determinant of it [45]. When patients report positive experiences, it often indicates that the care they received was empathetic, effective, and aligned with their expectations and needs. This aspect of healthcare is a crucial measure of quality, as it encompasses not only the clinical outcomes but also the process through which care is delivered. In the context of VBHC, quality, as reflected in patient experience, becomes a central attribute. VBHC emphasizes that the value of healthcare is determined not just by clinical efficacy but also by how well the care process meets patient expectations and improves their overall health journey. Thus, enhancing patient experience is a direct pathway to elevating the quality of care in VBHC. In the healthcare context, quality typically encompasses various virtues, focusing on adherence to established processes and inputs [2]. This emphasis on patient experience aligns clinical efficacy with personalized care delivery, reinforcing the dual mandate of VBHC to improve both outcomes and experiences. Research suggests that patient experience plays a critical role in adherence to medical regimens, reduced anxiety, and overall perceptions of care quality [46, 47]. Incorporating patient-reported experience measures (PREMs) alongside traditional clinical outcomes allows for a more comprehensive evaluation of healthcare interventions [23].

#### 3.2.3. PCC

A third attribute, integral to VBHC, that our analysis defined is PCC, which emphasizes the active engagement of patients in their care and treatment decisions. This attribute focuses on the inclusion of patients' preferences, beliefs, and values in all aspects of care delivery [26, 48]. Care is characterized to be patient-centered if patients are treated by providers as shared decision-makers, have autonomy in making informed decisions about treatment choices, and take into account their preferences [49].

The IOM identified PCC as a core competency for healthcare professionals, emphasizing its role in addressing quality gaps in healthcare systems. By shifting from a traditional, provider-centric approach to a patient-focused model, PCC becomes a vital driver of quality improvement in VBHC [39]. This transition requires decisive leadership in healthcare institutions to integrate patient-centered outcomes into organizational goals [50].

VBHC is designed to maximize healthcare resource utilization and assess the efficiency of health systems. It centers on the significance of medical treatment to the patient, specifically in terms of health outcomes as perceived and evaluated by the patients themselves [1]. Evidence-based medicine further supports this alignment by defining valuable outcomes as those that are significant and beneficial to individual patients [51]. For a complete alignment of VBHC and PCC, it is essential that patient-centered outcomes, viewpoints, and preferences are explicitly factored into the definitions and measures of quality, cost, and value [52]. These elements are increasingly pivotal in shaping the delivery of care. Thus, PCC ensures that VBHC not only addresses clinical outcomes but also elevates patient satisfaction and aligns care delivery with patients' unique needs and expectations.

In sum, the concept of VBHC is defined by three interrelated attributes: monetary value of health services, quality of care, and PCC. Together, these attributes reflect a healthcare model that seeks to optimize financial efficiency, improve clinical outcomes, and enhance the patient experience. While these three attributes of VBHC are interrelated, their relative importance can vary depending on stakeholder perspectives and healthcare system priorities. While some models of VBHC emphasize cost containment, others prioritize patient outcomes and experience, making trade-offs an inherent part of value determination.

A program that improves patient satisfaction but does not reduce costs or improve clinical outcomes may still hold value, particularly in patient-centered healthcare models. Research suggests that positive patient experiences enhance engagement, adherence to treatment, and trust in the healthcare system, which can indirectly lead to better health outcomes over time [40, 46]. In addition, in competitive healthcare markets, patient experience influences hospital reputation, patient retention, and financial performance, making it an economically relevant factor even if direct cost savings are not apparent [53].

A program that improves patient satisfaction without changing health outcomes is still aligned with the patient-centered dimension of VBHC. While traditional healthcare models prioritize clinical outcomes, emerging frameworks, such as the quadruple aim, explicitly recognize patient experience as a key metric of quality [54]. Satisfaction-driven improvements—such as better communication, reduced wait times, or enhanced provider empathy—may not always translate into measurable health gains but can significantly improve perceived quality and trust in healthcare, which are critical to PCC.

A program that improves health outcomes but at an increased cost can still align with VBHC principles if the clinical benefits justify the financial investment. VBHC does not mandate absolute cost reduction but rather cost-effectiveness—meaning that increased spending is acceptable if it leads to proportionally greater health gains [38]. Health economic models, such as incremental cost-effectiveness ratios (ICERs), are used to determine whether the additional cost is justified by improved outcomes. In cases where improved outcomes lead to long-term cost savings (e.g., through reduced hospitalizations or better chronic disease management), initial cost increases may be viewed as an investment rather than an inefficiency [55].

Ultimately, the salience of each VBHC attribute depends on stakeholder priorities, healthcare financing models, and the context in which the program is implemented. Some systems prioritize cost control, while others focus on patient-centered outcomes or clinical effectiveness. Rather than treating cost, quality, and patient experience as rigid trade-offs, VBHC should be viewed as a dynamic model that seeks to optimize all three dimensions in balance with stakeholder needs and healthcare goals.

Understanding the interplay between these attributes is essential for effectively integrating VBHC principles into healthcare systems, ensuring that both economic sustainability and patient well-being remain at the core of care delivery.

#### 3.2.4. Integration of Attributes

VBHC revolves around achieving better health outcomes for patients at lower costs while aiming for patient satisfaction. The integration of the three attributes—quality of care, value for money, and PCC— creates synergistic framework that supports this goal. For instance, high-quality care minimizes inefficiencies, reduces medical errors, and prevents unnecessary treatments. Thus, quality improvement efforts directly contribute to the economic sustainability of healthcare systems.

PCC is seen as a catalyst for better outcomes and patient satisfaction. PCC ensures that healthcare delivery aligns with the preferences, needs, and values of patients. This approach enhances communication, strengthens trust, and improves patient adherence to treatments. In addition, PCC reduces wasteful practices, such as unnecessary diagnostic tests or procedures, thereby contributing to overall cost savings in the healthcare system.

Finally, these attributes do not operate in isolation but reinforce one another. Quality of care improves outcomes, which is a fundamental component of VBHC. Simultaneously, PCC ensures that the delivery of high-quality care is both effective and efficient, leading to better patient experiences and cost savings. Together, these three attributes form a cohesive, synergistic framework for achieving the overarching goals of VBHC.

VBHC is best understood as an integrated framework that aligns PCC, healthcare quality, and cost-effectiveness rather than treating them as distinct or opposing elements. While previous discussions in the literature have sometimes framed these aspects in dualistic terms, VBHC fundamentally operates as a unified approach where improved patient outcomes are achieved through optimized healthcare delivery and efficient resource utilization. Outcome measurement in VBHC is not confined to a single dimension but instead considers multiple interdependent factors that collectively define value in healthcare. By incorporating cost-effectiveness, patient experience, and quality into a holistic evaluation, VBHC ensures a comprehensive understanding of healthcare impact.

### 3.3. Identifying Model, Borderline, and Contrary Cases

According to Walker and Avant's [12] model, a concept analysis needs to identify model, borderline, and contrary cases. The model case embodies all the attributes of the concept, the borderline case exhibits some but not all attributes, and the contrary case lacks the attributes altogether.

#### 3.3.1. Model Case

Alex, a 45-year-old high school teacher with a busy lifestyle, was recently diagnosed with Type 2 diabetes during a routine health screening. The diagnosis was unexpected and prompted Alex to reconsider his health priorities. He lives in a bustling urban area, where his demanding job and personal commitments leave him little time for regular exercise or meal planning.

Upon his diagnosis, Alex was quickly integrated into a diabetes care program designed around VBHC principles. The program assigned him a care team consisting of a primary care physician, a diabetes educator, a dietitian, and a digital health advisor. This multidisciplinary team met with Alex to develop a comprehensive care plan tailored to his unique needs, preferences, and lifestyle challenges. The plan focused on critical areas such as dietary changes, physical activity, and effective medication management.

To optimize the monetary value of health services for Alex, the healthcare system enrolled him in a digital health monitoring program. This innovative approach leveraged technology, including a smart glucometer and a mobile application, to track his blood sugar levels, diet, and exercise routine. The digital health advisor could remotely monitor Alex's progress, adjusting his care plan as necessary. This remote monitoring capability meant that Alex could receive adjustments to his care without the need for frequent, time-consuming in-person visits, thereby saving costs and making efficient use of healthcare resources.

The quality of care that Alex received was grounded in the latest evidence-based guidelines for managing Type 2 diabetes. His personalized care pathway ensured that every aspect of his treatment was aligned with current best practices, from medication management to lifestyle interventions. The centralized digital health record system used by his care team facilitated seamless communication and coordination among providers, ensuring that any adjustments to his care were made swiftly and based on a comprehensive view of his health status. His care team offered flexible consultation hours and utilized digital communication tools to fit his busy schedule. They engaged Alex in the decision-making process, respecting his preferences and involving him in crafting a treatment plan that was both effective and aligned with his values. The diabetes educator played a crucial role in providing Alex with practical advice for incorporating healthy habits into his daily routine, as well as offering emotional support to help him navigate the challenges of managing a chronic condition.

To further enhance PCC, Alex's care plan integrated patient-reported outcome measures (PROMs) to systematically assess his progress. Through periodic surveys and self-reported health assessments, Alex provided feedback on his physical well-being, daily functioning, and quality of life. This additional layer of patient input allowed his care team to make more informed adjustments to his treatment plan, ensuring that interventions were not only clinically effective but also aligned with his personal experiences and health priorities.

After 6 months in the program, Alex saw significant improvements in his glycemic control, as evidenced by lower HbA1c levels. He felt more energetic and was more confident in his ability to manage his diabetes. The convenience and cost savings associated with the digital health tools and fewer in-person visits were particularly valuable to Alex, enhancing his satisfaction with the care he received.

The case of Alex demonstrates the importance of personalized and comprehensive care in managing chronic conditions like Type 2 diabetes. By integrating him into a care program that focused on his unique needs and challenges, Alex was able to see significant improvements in his health. The use of digital health tools and remote monitoring allowed for more efficient and cost-effective care, which was particularly beneficial for someone like Alex with a busy lifestyle. This case highlights the potential of VBHC principles in improving patient outcomes and satisfaction. It also emphasizes the importance of involving patients in the decision-making process and providing them with practical advice and emotional support.

#### 3.3.2. Borderline Case

Jordan, a 50-year-old office manager, was diagnosed with Type 2 diabetes. Upon diagnosis, Jordan was introduced to a primary care framework aimed at managing his new chronic condition. He was assigned a primary care physician and a nurse practitioner specializing in diabetes management. This team, while knowledgeable and dedicated, represented a more conventional approach to care, lacking the broad, multidisciplinary collaboration and the robust digital health infrastructure.

Jordan's treatment plan incorporated cost-effective strategies, such as the prescription of generic medications and standard dietary consultations aimed at managing his diabetes. This approach demonstrated a certain awareness of the need to control healthcare costs, yet it did not fully exploit the potential efficiencies of digital health technologies. Without a comprehensive digital monitoring system, Jordan found himself required to make frequent, often inconvenient, in-person visits to the clinic for routine blood sugar checks and follow-up consultations. This necessity for physical visits underscored a partial commitment to the monetary value aspect of VBHC, recognizing cost considerations but not fully addressing efficiency and convenience through technology.

The quality of care that Jordan received adhered to established guidelines for diabetes management, ensuring a basic level of effectiveness and safety. However, the absence of a cohesive, integrated care team and the limited use of advanced health information technology constrained the system's ability to dynamically adjust Jordan's care in response to real-time data. As a result, changes to his treatment plan tended to be reactive, based on periodic assessments rather than ongoing monitoring, somewhat diminishing the overall impact of his care.

In terms of PCC, Jordan's providers did make efforts to accommodate his needs and preferences within the constraints of their system. Jordan had some opportunity to voice his concerns and influence his care plan, yet the lack of a comprehensive support infrastructure—such as a dedicated diabetes educator and flexible digital communication tools—meant that his engagement with his care was more limited than it could have been. The system's partial adoption of VBHC principles meant that while Jordan's care was somewhat personalized, it lacked the depth of patient engagement and the customization seen in fully realized VBHC models.

Jordan saw moderate improvements in managing his diabetes, appreciating the cost-conscious aspects of his care and the adherence to quality guidelines. Yet, he could not help feeling that his care experience could have been more effective and personalized. The frequent clinic visits felt inefficient and sometimes unnecessary, highlighting a gap between the ideal of VBHC and the reality of a system only partially aligned with its values.

Jordan's experience highlights the limitations of a healthcare system that only partially adopts the values of VBHC. While his care was cost-effective and adhered to quality guidelines, the lack of a comprehensive digital monitoring system and a multidisciplinary care team limited the effectiveness and personalization of his care. Frequent in-person visits and limited patient engagement underscore the need for a more integrated approach that fully utilizes digital health technologies and prioritizes PCC. Jordan's case emphasizes the importance of aligning healthcare systems with the principles of VBHC to optimize patient outcomes and experiences.

#### 3.3.3. Contrary Case

Sam, a 60-year-old retiree, had chronic obstructive pulmonary disease (COPD). Sam's journey is symbolic of a traditional fee-for-service healthcare system, where the emphasis is on the quantity of services provided rather than their quality or the outcomes they produce.

Sam's interactions with his healthcare providers are marked by a series of frequent hospital admissions, alongside an exhaustive array of diagnostic tests and procedures. This approach to healthcare, while extensive, lacks the coordinated effort necessary for effective chronic disease management. The primary aim seems to be addressing acute issues as they arise, without a comprehensive, proactive plan for managing Sam's COPD over the long haul. The system's focus on immediate interventions, without a significant consideration of their long-term value or cost-effectiveness, leads to a fragmented care experience that does little to improve Sam's overall health or well-being. Within this healthcare model, discussions about the cost-effectiveness of treatments are noticeably absent. Decisions are made with an eye toward immediate action, putting aside considerations of whether these actions offer the best outcomes for the investment made. This lack of transparency and consideration for costs results in Sam and his family facing unexpected and often high medical bills, underscoring a system more concerned with volume than value.

The quality of care that Sam receives is inconsistent at best. While he encounters instances of excellent care from individual providers, the overall system's disinterest in coordinated care and quality outcomes leads to a care experience that varies widely in effectiveness. Opportunities for preventive measures and education, which could significantly improve Sam's condition and reduce his reliance on acute care, are frequently overlooked. Moreover, Sam's role in the management of his COPD is largely passive. The system does not prioritize patient engagement or make efforts to tailor care to his specific needs and preferences. This oversight fosters a sense of disconnection between Sam and his care providers, leaving him feeling more like a recipient of services rather than an active participant in his healthcare journey.

As a result of this volume-focused approach, Sam's health management is far from optimal. He finds himself caught in a cycle of emergency room visits and hospitalizations that address his COPD symptoms without ever truly improving his condition or quality of life.

This case reflects the shortcomings of a traditional fee-for-service healthcare system The system focuses on providing a large quantity of services rather than prioritizing their quality or outcomes. Sam's care is marked by frequent hospital admissions and diagnostic tests but lacks coordination and a proactive plan for managing his chronic disease. The system's emphasis on immediate interventions without considering long-term value or cost-effectiveness leads to fragmented care and high medical bills. The quality of care Sam receives is inconsistent, and opportunities for preventive measures and patient engagement are often overlooked. As a result, Sam's health management is suboptimal, with his condition and quality of life not improving.

### 3.4. Identifying Antecedents and Consequences

In the model presented by Walker and Avant [12], the seventh step involves pinpointing antecedents and consequences. Antecedents pertain to the events or situations that need to happen before a concept can take place. On the other hand, consequences are the events or situations that arise because of the realization of the concept.

#### 3.4.1. Antecedents

In the context of implementing VBHC, several antecedents are crucial for the successful transition from traditional fee-for-service models to those focused on value. The antecedents can be understood as the conditions or factors necessary for the adoption and effective implementation of VBHC.

One primary antecedent is the availability of comprehensive healthcare data, which enables the measurement and analysis of outcomes and costs associated with healthcare services [24]. The ability to track and analyze patient outcomes relative to the costs of care is foundational to VBHC, as it allows for the identification of value-creating practices. Healthcare systems must invest in robust IT infrastructure and data analytics capabilities to collect, store, and analyze health data effectively [56].

Another critical antecedent is supportive healthcare policy and reimbursement models that incentivize value over volume [1]. At the macro level, government and policymakers play a pivotal role in shaping the healthcare landscape through regulations, payment models, and incentives that encourage the adoption of VBHC principles. Policies that promote pay-for-performance, bundled payments, and accountable care organizations are examples of how healthcare systems can be incentivized to focus on value [57]. At the micro level, leadership within healthcare organizations is equally important. Leaders must champion the shift toward VBHC, fostering a culture that values continuous improvement, innovation, and PCC [58]. This involves not only strategic vision but also the operational alignment of incentives, processes, and stakeholder engagement to support VBHC implementation. It also requires transformational leadership to guide the shift to VBHC [7].

Stakeholder engagement, encompassing patients, healthcare providers, payers, healthcare managers, and policymakers, is another vital antecedent [24, 51]. The transition to VBHC requires a collaborative effort, where all stakeholders are aligned in their goals to improve health outcomes and create value. Engaging patients as active participants in their care, aligning provider incentives with health outcomes, and collaborating with payers and policymakers to support value-based models are essential steps in this process.

#### 3.4.2. Consequence

The implementation of VBHC leads to a series of consequences that significantly impact the healthcare system. These consequences include improved patient outcomes, cost reductions, and increased patient satisfaction. These consequences are not only interrelated but also reinforce the shift toward a more sustainable, efficient, and patient-centered healthcare system.

Improved patient outcomes are a direct consequence of adopting VBHC principles, as the focus shifts from the volume of services provided to the quality and effectiveness of care [24]. By prioritizing treatments and interventions based on their ability to deliver meaningful improvements in health, VBHC encourages healthcare providers to adopt evidence-based practices that are known to yield better health outcomes. Research has demonstrated that systems oriented toward value can lead to enhancements in patient health, including reduced mortality rates, lower readmission rates, and improved management of chronic diseases [59].

Cost reduction is another significant consequence of VBHC. As healthcare systems become more focused on delivering value, there is a natural progression toward eliminating waste, such as unnecessary tests or ineffective treatments, and optimizing resource allocation [1]. This efficiency not only benefits patients by reducing their out-of-pocket expenses but also alleviates the financial burden on the entire healthcare system, making it more sustainable in the long run [60].

Increased patient satisfaction is a further consequence of implementing VBHC. When care is patient-centered, transparent, and tailored to individual needs, patients are more likely to be engaged in their treatment and satisfied with their care experience [51]. VBHC's emphasis on patient outcomes and involvement in care decisions fosters a stronger patient-provider relationship, enhancing trust and satisfaction. Moreover, the holistic approach to care, considering the patient's overall well-being rather than just acute interventions, contributes to higher levels of patient satisfaction [61].

#### 3.4.3. Defining Empirical Referents

The final step in analyzing the VBHC concept is to identify empirical referents for the defining attributes [12]. Empirical referents are observable indicators that demonstrate the occurrence of the concept in the real world, serving as a bridge between its abstract conceptual understanding and its practical measurement. In the context of VBHC, empirical referents help in assessing how well the principles of VBHC are understood, implemented, and their impact on healthcare delivery and outcomes.

To measure VBHC in practice, its three defining attributes—monetary value of health services, quality of care, and PCC—must be assessed using empirical referents. These indicators provide tangible ways to determine the extent to which VBHC principles are implemented in healthcare systems.

The monetary value of health services can be evaluated through cost-effectiveness and financial efficiency measures. For instance, the ICER assesses whether additional spending yields better outcomes [38], while total cost of care (TCOC) evaluates per-patient costs across all services [57]. Payment models such as bundled payments encourage cost control by tying reimbursement to entire episodes of care rather than individual services [62]. In addition, avoidable hospitalizations and readmission rates signal inefficiencies, highlighting areas where costs and outcomes could be better aligned [63]. These measures ensure that VBHC optimizes both financial sustainability and care quality.

The quality of care component can be assessed through both patient-reported and clinical indicators. PROMs capture functional improvements and symptom relief [64], while clinical indicators such as mortality, infection, and complication rates provide objective measures of care effectiveness [65]. PREMs are also important in measuring patients' experience in the process of receiving care [66] Standardized tools like the Healthcare effectiveness data and information set (HEDIS) track evidence-based care delivery [67], and adherence to clinical guidelines ensures providers follow best practices [1]. In addition, length of hospital stay and recovery time serve as indirect measures of efficiency and care quality [55]. Together, these referents provide a comprehensive evaluation of healthcare quality under VBHC.

The PCC aspect of VBHC can be measured through indicators capturing patient experience and engagement. PREMs assess communication quality, provider responsiveness, and overall satisfaction [46]. Tools such as the shared decision-making process (SDMP) scale scores gauge patient involvement and provider trust [68]. The patient-centered innovation questionnaire nurses could use to evaluate the implementation of PCC [69]. In ddition, the consumer assessment of healthcare providers (CAHPS) surveys provide standardized assessments of patient satisfaction [28], while the continuity of care index (COCI) measures the consistency of patient-provider relationships, which strengthens trust and engagement [70]. These referents ensure that VBHC prioritizes not just clinical outcomes but also the patient's healthcare experience.

By integrating financial, clinical, and experiential indicators, these empirical referents provide a structured way to measure VBHC's presence and effectiveness in real-world healthcare settings. Given Walker and Avant's [12] observation that empirical referents often mirror defining attributes, these measures serve as concrete tools to evaluate VBHC from multiple dimensions.

#### 3.4.4. Conceptual Model

**Figure 1:**
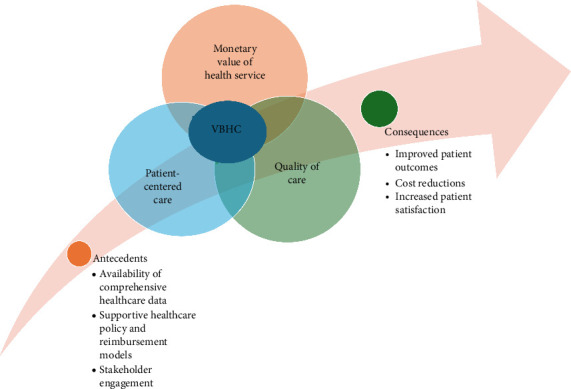
The model is a diagrammatic representation of the concept of VBHC.

Based on this conceptual analysis, we constructed a conceptual model for VBHC. The model sets out its key attributes, its antecedents and consequences, and empirical referents. The analysis also helped construct a model, borderline, and contrary cases which are integral to the concept-based analysis approach of Walker and Avant [12]. The VBHC concept model is shown in Figure 1.

This model offers a clear roadmap for healthcare decision-makers. By aligning programs with its three core attributes—monetary value, PCC, and quality—stakeholders can ensure critical prerequisites (like data, policies, and engagement) are in place and anticipate improved outcomes, lower costs, and greater patient satisfaction. It supports the design of new programs, the evaluation of existing ones, comparisons among competing options, and effective resource allocation. Overall, the diagram translates VBHC principles into a structured framework for real-world implementation.

## 4. Discussion

In this paper, we have conducted a detailed analysis of the concept of VBHC using Walker and Avant's concept analysis model [12]. The concept analysis illustrates the multifaceted nature of VBHC, underscoring its potential to transform healthcare delivery by aligning healthcare outcomes with patient values and reducing costs. The defining attributes of monetary value, quality of care, and PCC highlight the core components necessary for implementing VBHC. These attributes emphasize the importance of integrating patient perspectives into healthcare delivery, ensuring care is both high in quality and economically efficient.

The model, borderline, and contrary cases provide concrete examples of how VBHC principles can be applied in practice, illustrating the variability in implementation and outcomes.

The analysis underscores the need for healthcare systems to embrace VBHC as a strategic priority. It requires a deep-seated change in the way organizations operate, focusing on enhancing patient outcomes while also improving cost-effectiveness [71]. The leadership and management within healthcare are identified as crucial agents of this change, requiring them to possess a broad spectrum of knowledge, skills, and attitudes to effectively manage the intricacies involved in the adoption of VBHC [72]. Moreover, there is a pronounced need for specific educational and training initiatives aimed at providing healthcare workers with the essential skills needed for the successful application of VBHC principles [73].

The concept analysis identifies a significant knowledge gap within the literature regarding the operationalization and measurement of VBHC. Despite the growing interest in VBHC, there is a lack of uniform standards and empirical evidence on its effectiveness across different healthcare settings. This gap presents a critical opportunity for future research to develop and validate standardized tools for measuring the impact of VBHC on patient outcomes and cost efficiency. Moreover, exploring the patient perspective on VBHC implementation can provide valuable insights into the quality of care and patient satisfaction, further informing the development of PCC models.

The antecedents and consequences identified in the analysis highlight the importance of supportive healthcare policies and reimbursement models that incentivize value over volume. Policymakers and healthcare leaders must collaborate to create an enabling environment for VBHC, including the development of alternative payment models and investment in healthcare IT infrastructure. Such policies can facilitate the transition toward a more sustainable and efficient healthcare system that aligns with the United Nations' Sustainable Development Goals.

The concept analysis of VBHC lays the groundwork for a deeper understanding of its implications for healthcare delivery and policy. Future research should focus on longitudinal studies to evaluate the long-term effects of VBHC on healthcare outcomes and cost savings. In addition, exploring the implementation challenges and success factors across diverse healthcare contexts can provide valuable lessons for scaling VBHC principles globally. Engaging a broader range of stakeholders, including patients, families, healthcare providers, and policymakers, in the research process can enhance the relevance and applicability of findings.

From a system perspective, VBHC provides a structured approach to guiding health systems in their budget allocation and funding decisions. Healthcare systems constantly face challenges in determining which programs to sustain, expand, or discontinue. By applying VBHC principles at the macro level, decision-makers can prioritize funding for programs that demonstrate measurable improvements in patient outcomes, cost efficiency, and care quality. This ensures that investments align with long-term healthcare sustainability goals and drive high-value interventions. A VBHC framework offers a data-driven methodology to support strategic resource distribution, ensuring that funding is directed toward initiatives that yield the greatest benefit for both patients and healthcare systems. This broader application of VBHC can inform not only service delivery but also high-level policy and financial decisions, reinforcing its role as a fundamental driver of efficient healthcare investment.

Based on this concept analysis, VBHC can be defined “as a patient-centered healthcare delivery model that aims to maximize the value of services provided to patients by focusing on improving health outcomes and optimizing the cost-effectiveness of care. VBHC is characterized by the strategic alignment of healthcare practices, policies, and stakeholder engagements to prioritize quality, efficiency, and patient satisfaction. Through the integration of comprehensive data analytics, supportive policies, and collaborative stakeholder involvement, VBHC seeks to transform the healthcare system into one that rewards value over volume, thereby ensuring sustainable improvements in patient health while simultaneously reducing unnecessary healthcare expenditures”.

## 5. Strengths and Limitations

This concept analysis on VBHC presents several strengths and limitations that merit consideration. Among its strengths, the analysis provides a comprehensive and systematic exploration of the VBHC concept, employing the Walker and Avant framework to dissect its defining attributes, antecedents, consequences, and empirical referents. This methodical approach ensures a thorough understanding of VBHC, highlighting its potential to revolutionize healthcare delivery by emphasizing PCC, quality, and cost-efficiency. Furthermore, the inclusion of model, borderline, and contrary cases enrich the analysis by offering practical insights into how VBHC principles can be applied in real-world settings, thereby illustrating the variability in implementation and potential outcomes.

However, the analysis also has certain limitations. One notable limitation is the reliance on existing literature, which may not fully capture the rapidly evolving landscape of VBHC implementation across different healthcare systems. This could lead to a knowledge gap regarding the latest challenges and innovations in VBHC. In addition, while the analysis attempts to address the conceptual and operational definitions of VBHC, it may not sufficiently account for the diverse interpretations and applications of VBHC in various healthcare contexts, potentially limiting its generalizability.

## 6. Implications for Nursing Management

The concept analysis of VBHC has significant implications for nursing management, particularly in enhancing leadership, optimizing resource allocation, and improving PCC. Nurse managers play a crucial role in integrating VBHC principles into daily practice by fostering a culture of continuous improvement, evidence-based decision-making, and cost-effective care delivery. Effective leadership in nursing management involves aligning workflows with VBHC objectives, advocating for policies that support value-based care, and ensuring adherence to quality and safety standards.

Workforce development is essential for the successful implementation of VBHC. Nurse managers must support ongoing education and training programs that equip nurses with the skills necessary to provide high-quality PCC while being mindful of resource utilization. Integrating VBHC principles into nursing curricula and professional development initiatives enhances the ability of nursing staff to measure and improve patient outcomes.

Operational efficiency is another critical area where nursing management influences the adoption of VBHC. Efficient staffing models, streamlined workflows, and data-driven decision-making help reduce inefficiencies without compromising care quality. The use of health information technologies, such as electronic health records and telehealth, facilitates better care coordination and enhances the ability to track patient outcomes, manage chronic conditions, and reduce unnecessary hospital admissions.

A fundamental aspect of VBHC is patient engagement, and nursing managers must promote strategies that foster meaningful interactions between nurses and patients. Encouraging shared decision-making, integrating PROMs, and prioritizing effective communication enhance patient satisfaction and adherence to treatment plans. In addition, interdisciplinary collaboration is vital in ensuring that nursing teams work closely with other healthcare professionals to provide holistic, coordinated care.

## 7. Conclusion

In conclusion, the concept analysis of VBHC provides a comprehensive framework for understanding its key components, implications, and challenges. By aligning healthcare delivery with the values and needs of patients, VBHC represents a promising avenue toward achieving high-quality, sustainable healthcare. The findings from this analysis call for a collaborative effort among healthcare professionals, researchers, and policymakers to further refine and implement VBHC models, ensuring healthcare systems are both patient-centered and cost-effective. As the healthcare landscape continues to evolve, VBHC offers a roadmap for navigating the complexities of modern healthcare delivery, aiming to enhance patient outcomes and achieve economic sustainability.

## Data Availability

The data that were used to support this concept analysis can be found within the texts of the cited references included in this paper.
